# The Other Dimension—Tuning Hole Extraction via Nanorod Width

**DOI:** 10.3390/nano12193343

**Published:** 2022-09-25

**Authors:** Tal Rosner, Nicholas G. Pavlopoulos, Hagit Shoyhet, Mathias Micheel, Maria Wächtler, Noam Adir, Lilac Amirav

**Affiliations:** 1 Schulich Faculty of Chemistry, The Russell Berrie Nanotechnology Institute, The Nancy and Stephen Grand Technion Energy Program, Technion−Israel Institute of Technology, Haifa 32000, Israel; 2 Department Functional Interfaces, Leibniz Institute of Photonic Technology Jena, Albert-Einstein-Straße 9, 07745 Jena, Germany; 3 Institute of Physical Chemistry and Abbe Center of Photonics, Friedrich Schiller University Jena, Helmholtzweg 4, 07743 Jena, Germany

**Keywords:** semiconductor nanorods, seeded rods, photocatalysis, hydrogen, transient absorption, hole extraction

## Abstract

Solar-to-hydrogen generation is a promising approach to generate clean and renewable fuel. Nanohybrid structures such as CdSe@CdS-Pt nanorods were found favorable for this task (attaining 100% photon-to-hydrogen production efficiency); yet the rods cannot support overall water splitting. The key limitation seems to be the rate of hole extraction from the semiconductor, jeopardizing both activity and stability. It is suggested that hole extraction might be improved via tuning the rod’s dimensions, specifically the width of the CdS shell around the CdSe seed in which the holes reside. In this contribution, we successfully attain atomic-scale control over the width of CdSe@CdS nanorods, which enables us to verify this hypothesis and explore the intricate influence of shell diameter over hole quenching and photocatalytic activity towards H_2_ production. A non-monotonic effect of the rod’s diameter is revealed, and the underlying mechanism for this observation is discussed, alongside implications towards the future design of nanoscale photocatalysts.

## 1. Introduction

The development of clean and renewable energy sources is vital for the preservation of modern society in light of the impending energy crisis and the potential for environmental catastrophes such as global warming. Solar-to-hydrogen generation is a promising approach to generate clean and renewable fuel. Nearly perfect 100% photon-to-hydrogen production efficiency for the photocatalytic water splitting reduction half reaction was already demonstrated utilizing a nanoscale artificial photosynthetic system based on a platinum-tipped CdS rod with an embedded CdSe seed [1]. This hybrid architecture facilitates efficient long-lasting charge-carrier separation, as the delocalized electrons are transferred easily to the metal tip, while the holes are three-dimensionally confined to the CdSe seed [2,3,4]. However, this CdSe@CdS-Pt nanorod structure is not suitable for overall water splitting as it is hampered by photochemical instability and requires sacrificial electron donors. The key bottleneck for efficient hydrogen evolution from such semiconductor nanorods, and their long-term stability, was proven to be the rate at which photoexcited holes are extracted [2,5,6,7,8]. Recently it was further demonstrated that the challenges which must be overcome in order to realize overall water splitting and O_2_ production are of kinetic rather than of a thermodynamic nature [9,10]. Hence, facilitating efficient hole transfer is a critical aspect in the design of improved CdS-based photocatalytic systems.

The majority of attempts in the literature which strived to facilitate hole extraction focused on the acceptor side and examined coupling the semiconductor particles with hole-acceptor entities spanning from metal oxides [11,12,13] to ligands and organic coatings [14,15,16], as well as molecular catalysts [17,18,19]. Several reports suggested that hole extraction efficiency might be sensitive to the rod’s dimensions [20,21,22,23,24,25]. This, in turn, implies the possibility to improve hole extraction directly via proper design of the photocatalytic system. One interesting approach along this line was proposed by Zamkov and coworkers, who suggested that an ultra-thin CdS shell would enable improved extraction of holes from the CdSe core [22]. The reduced shell thickness in CdSe@CdS nanorods (NRs) was achieved via slow etching of the CdS shell, and this resulted in an increased rate of hole extraction, leading to ~3–4-fold increase in photocatalytic H_2_ generation. This work clearly demonstrates the potential of improving hole extraction by tuning the rod’s width, yet the precise effect of thickness on hole extraction and catalytic activity is still not fully explored. Furthermore, etching is challenging to accurately control, and the method may introduce surface defects that can serve as charge traps and reduce the photocatalytic activity.

Here, we directly synthesize nearly monodispersed CdSe@CdS NRs of varying widths and demonstrate the non-monotonic effect of the rod’s diameter on hole extraction efficiency and photocatalytic hydrogen evolution activity. The underlying mechanism for the observed trends is discussed alongside implications towards future design of nanoscale photocatalysts.

## 2. Results and Discussion

**Figure 1:**
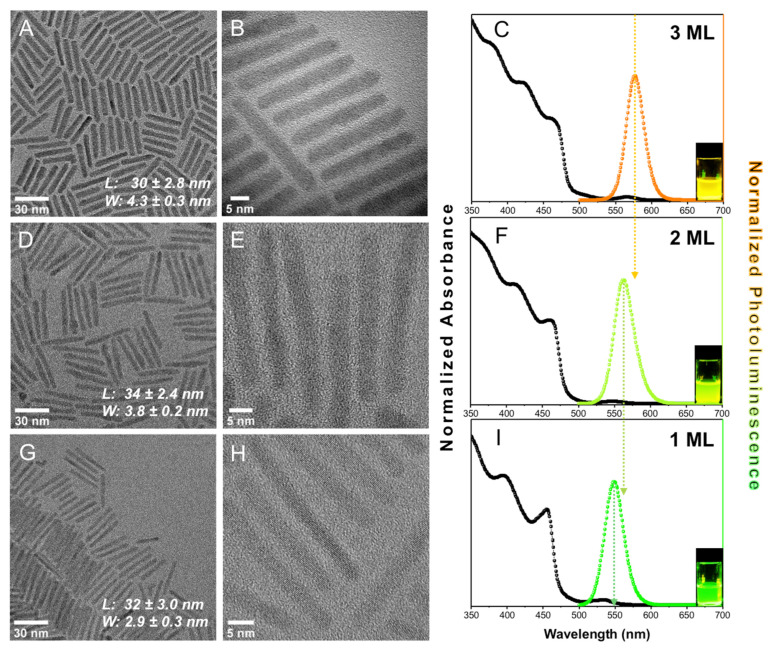
Optical characterizations of nanomaterials. TEM and UV-Vis absorption and PL spectra of 3 ML (**A**–**C**), 2 ML (**D**–**F**), and 1 ML (**G**–**I**) NRs in toluene, excitation wavelength 405 nm. The letters “L” and “W” in panels (**A**,**D**,**G**) are abbreviations for “length” and “width”, respectively. Insets in panels (**C**,**F**,**I**) show the luminescence of the different nanoparticles in solution with a glass vial.

**Synthesis and Characterization of CdSe@CdS Nanorods**. Colloidal CdSe@CdS NRs were synthesized according to well-known protocols [26,27,28]. Figure 1 A,B,D,E,G,H depict TEM images of NRs that are all ~30 nm in length and have widths of 2.9 ± 0.3, 3.8 ± 0.2, and 4.3 ± 0.3 nm, respectively (additional statistics can be found in the SI Figure S1). By taking into account the size of the CdSe core (d = 2.3 nm) and by knowing that a monolayer (ML) of CdS shell is 0.35 nm, the NRs are calculated to have 1, 2, and 3 ML of CdS, respectively [29]. This demonstrates that precise atomic-scale control over the width of the CdSe@CdS NRs under constant CdSe core size was successfully attained. Variation in the NR width was achieved by slight modifications to the crystal structure and concentration of the CdSe seeds used for the synthesis of CdSe@CdS NRs (see supporting information for details).

The NRs were further characterized by absorption and photoluminescence (PL) spectroscopy (Figure 1C,F,I). The absorption spectra below 500 nm are related to the 1Σ (1σ_e_-1σ_h_) and 1Π (1π_e_-1π_h_) excitonic transitions in the CdS shell [30,31]. The weaker absorption feature above 500 nm is assigned to the lowest energetic 1Σ (1σ_e_-1σ_h_) transition in the CdSe core. Further, minor contributions of an excitonic CdS transition stemming from the CdS shell surrounding the CdSe seed, which is slightly redshifted compared to the CdS rod 1Σ transition, is hidden in the red shoulder of the CdS rod band edge transition [30]. As the nanorod thickness increases from 1–3 ML, the positions of the CdS excitonic transition (1–3 ML: ~456–462 nm, ΔE = 0.04 eV), the CdSe excitonic transition (1–3 ML: ~530–568 nm, 0.16 eV), and the photoluminescence (1–3 ML: ~548–577 nm, 0.11 eV) band red-shift. This occurs as a result of reduced confinement with growing rod width and increasing electron delocalization from the CdSe core into the CdS shell [32].

**Photocatalytic hydrogen evolution reactions** were performed to study the catalytic activity of the 1, 2, and 3 ML NRs. The photocatalytic activity towards H_2_-production utilizing nanorods was previously found as sensitive to the metal tip size, and potentially also the contact area [33]. Given the variation in rod widths, attaining the desired level of control over the Pt domain size and interface diameter was beyond our reach. In order to enable proper comparison between the various rods, the metal cocatalyst in this work had to be decoupled from the rods. Hence, Pt nanoparticles that were freely mixed with the rods were utilized, with methyl viologen (MV^2+^; 4.5 mM) acting as an electron shuttle that transferred electrons from the photosensitized rods to the Pt nanoparticles (Pt-NPs; 0.8 mM), on which the reduction reaction took place [34]. 3-mercaptopropionic acid (MPA; 0.11 M) was used as both a water-dispersible ligand and as a hole scavenger. For further details, the reader is referred to the SI. The solution was dispersed in a phosphate buffer (pH 6.2, 50 mM) and placed inside of a custom-built gas tight reaction cell (details of which were published previously) [1]. The concentration of nanorods was adjusted to an optical density of 2.0 at 405 nm (~5–10 × 10^14^ rods per sample, see SI for detailed explanation on sample absorbance adjustments and Figure S2 for absorption spectra of NRs and Pt-NPs). The cell was purged with argon for one hour and then irradiated at 5 mW with a 405 nm LED to excite the rods. The evolved hydrogen was measured using an online gas chromatograph equipped with a thermal conductivity detector. Operation in continuous flow mode allowed for direct determination of the gas production rate. The internal quantum efficiency of the sample, which is defined as QE = 2N_H2_/_absorbed-_N_hυ_, was determined by quantifying the amount of evolved hydrogen at a photon flux of 5.09 × 10^15^ photons s^−1^ (see supporting information for details on the calculations).

**Figure 2:**
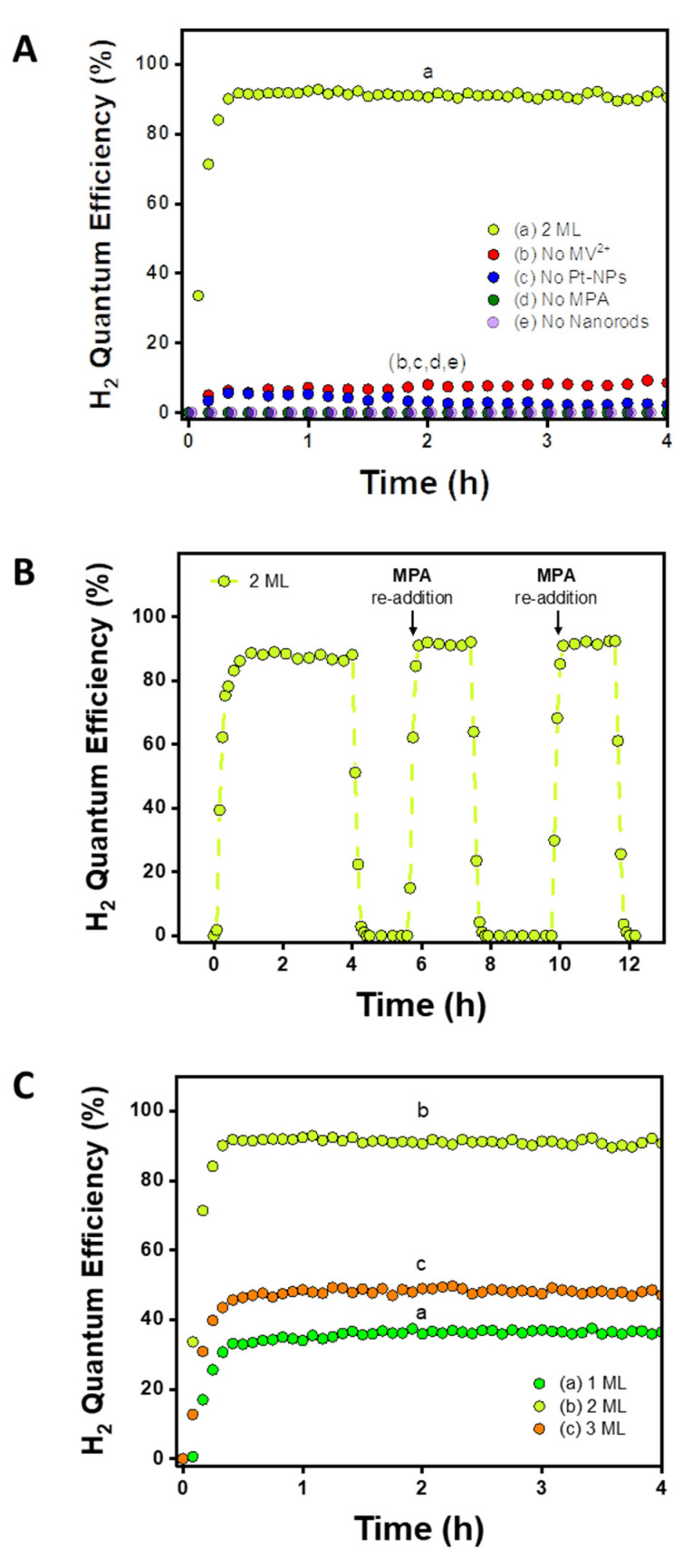
Photocatalytic H2 evolution reactions. (**A**) Control experiments where MV^2+^, Pt-NPs, MPA, and 2 ML NRs are omitted independently. Solutions were irradiated at 405 nm. Conditions unless stated otherwise: NR absorbance set to 1 at 405 nm, 4.5 mM MV^2+^, 136 mM MPA, 0.8 mM Pt-NPs, 50 mM pH 6.2 phosphate buffer, 5 mW. (**B**) 2 ML nanorods irradiated at 405 nm with re-addition of the hole scavenger (MPA). (**C**) Comparing the activity of 1, 2, and 3 ML NRs, irradiated at 405 nm.

Two control experiments were conducted to validate the proposed photocatalysis cycle. For these control experiments, we focus on the mid-range 2 ML sample, which serves as a representative case. In the first control experiment (Figure 2A), each species was independently omitted. In the absence of any of the components—MV^2+^ (redox shuttle), Pt-NPs (H_2_ evolving cocatalyst), MPA (hole scavenger), and NRs (photosensitizer)—the efficiency dropped substantially. This demonstrates that the system relies greatly on each component and corroborates the successful functionality of the shuttle-mediated photocatalytic system. In the second control experiment (Figure 2B), the reaction was run continuously for the first 4 h. Afterwards, the LED was turned off, an additional aliquot of MPA was added, and the LED was turned back on after proper purging of the reaction cell. Repeated iterations showed that the efficiency never exceeds unity (IQE_max_ = 92%). Moreover, continuously switching the LED off/on led to complete loss/recovery of the efficiency, validating the stability of the sample and demonstrating that the reaction was light-activated. Figure S3 presents 24 h operation without replenishment of the hole scavenger.

The H_2_ QE trend follows the order of 2 ML > 3 ML > 1 ML (Figure 2C). The 2 ML NRs showed considerably higher QE than the 1 and 3 ML NRs, displaying an impressive efficiency of 92% (under 405 nm excitation). These trends were highly reproducible and were obtained with several different sets of 1-2-3 ML rods (see Figure S4 for another set under 455 nm excitation).

**Monitoring Hole Extraction.** The trend for photocatalytic activity by which 2 ML outperforms 3 ML is in accordance with Zamkov and coworkers’ work and our original aim to improve extraction of holes from the CdSe core via a reduced CdS shell thickness. However, the observation that the 2 ML samples also improve upon the activity of the ultra-thin 1 ML samples calls for further examination. Thus, we first set out to verify the exact role and significance of hole extraction from the NRs in determining the relative activity of the three sample types. For this task, PL quenching studies were conducted using the commonly studied hole-quencher, phenothiazine (PTZ) [35]. A solution of NRs (adjusted to an absorbance of ~0.05 at the CdSe excitonic transition) was mixed with PTZ in toluene and excited at 415 nm.

**Figure 3:**
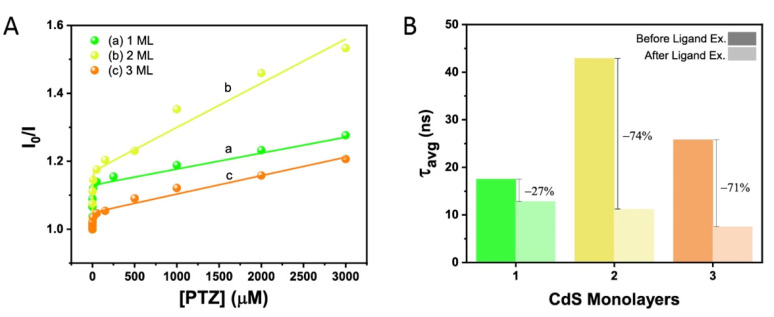
PL quenching experiment probing hole-transfer capabilities of the NRs. (**A**) Stern–Volmer plot of PL quenching in the presence of increasing amounts of PTZ as a hole scavenger in toluene. I_0_ and I are the PL intensity in the absence and presence of PTZ, respectively. (**B**) Average PL lifetime of NRs with native ODPA and with MPA ligands measured in water as solvent.

PL intensity began to decrease due to hole transfer to the PTZ quencher and the loss of radiative recombination in the NRs (Figure S5D,E). For example, with 3.0 mM PTZ (the highest concentration of PTZ used in the quenching experiment), the PL of the 1, 2, and 3 ML NRs was quenched by 22%, 35%, and 17%, respectively. The degree of quenching hence follows the order of 2 ML > 1 ML > 3 ML. To gain deeper insight into the interaction between the NRs and PTZ, the data was analyzed applying the Stern–Volmer formalism (Figure 3A). The Stern–Volmer Plot strongly deviates from a simple linear behavior and shows for all samples a region at low PTZ concentration with a very steep slope and, at higher PTZ concentration, a region with a lower slope. Such behavior is often observed in cases of either mixed static and dynamic quenching or when two different types of binding sites are present with different affinity for adsorption of the quencher or different accessibility. The data were appropriately fitted to a multi-site binding model [36] which has been used to describe nanorods [37,38] (for details, see SI Table S1). It is noteworthy that holes were more readily quenched for the 2 ML rods, in accordance with the activity for hydrogen production, despite the variation in experimental conditions such as the solvent, ligands, and hole scavenger. This indicates that this observed enhanced activity might indeed be attributed to the dimensions of the rods.

In order to probe the population of surface traps as a function of rod diameter, PL lifetime measurements were performed. The hydrogen evolution reactions were conducted with MPA acting both in the role of a ligand on the NR surface and as the hole scavenger. In addition, MPA is reported to induce electron traps which can be populated on the ns timescale and are responsible for changes in the PL lifetime [39,40]. Hence, the average PL lifetime of the 1, 2, and 3 ML MPA-capped NRs was ascertained and compared with that of the rods with native ODPA ligands. The average lifetime of ODPA-capped 1, 2, and 3 ML rods was measured to be of 17.5, 42.9, and 25.8 ns, respectively, while that of the MPA-caped NRs were measured to be 12.8, 11.2, and 7.5 ns, respectively (Figure 3B and Figure S6). The changes were calculated to be 4.7, 31.7, 18.3 ns, i.e., 27, 74, and 71%, for the 1, 2, and 3 ML rods, respectively. The more pronounced decrease in the average lifetime after ligand exchange to MPA that was obtained for the 2 ML and 3 ML samples may indicate that these rods had superior electron delocalization within the CdS shell relative to the 1 ML NRs, which increased the accessibility of electron traps located at the surface. This agrees with the expected quasi-type II band alignment of 2 ML and 3 ML usually observed in seeded nanorods with a seed size of 2.3 nm. In contrast, the comparatively short PL lifetime of 1 ML, which is an indicator of a fast rate for radiative recombination [41], and the smaller influence of the ligand exchange on the lifetime [42] hint towards a potential change in the band alignment of 1 ML from quasi-type II to type I.

**Transient Absorption.** To gain further insight into the electronic structure of the three different investigated samples, we recorded transient absorption spectra at different excitation wavelengths. Samples were excited with sub-100-fs pulses either at the CdS rod (λ_ex_ = 390 nm) or at the CdSe seed (λ_ex_ = 540 (1 ML), 560 (2 ML), or 570 nm (3 ML)) and probed with a white-light continuum (350–750 nm). All transient spectra shown were obtained at a delay time of 20 ps, i.e., after the initial fast processes of hole localization to the CdSe seed and the subsequent electron localization towards the seed had already taken place [30,33,43,44].

**Figure 4:**
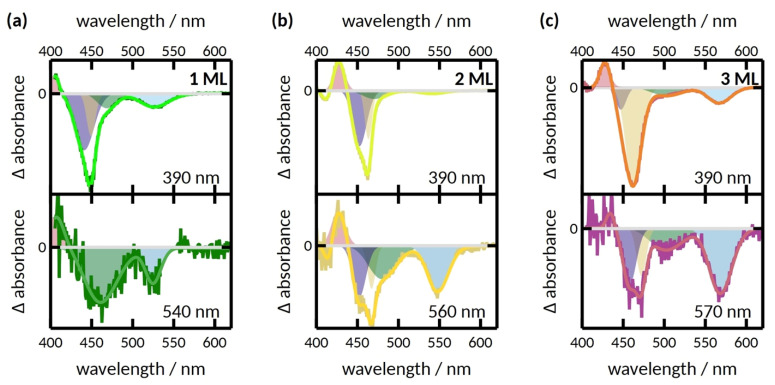
Transient spectra obtained at a delay time of 20 ps upon CdS rod (top) or CdSe seed (bottom) excitation of (**a**) 1 ML, (**b**) 2 ML, and (**c**) 3 ML. A multi-Gaussian fit to accurately describe the spectra is shown as shaded areas and the cumulative fit is shown as a solid line.

Before comparing spectra obtained at different experimental conditions, the features in the transient absorption spectrum of 1 ML upon CdS rod excitation are discussed. The spectrum is mainly characterized by three pronounced bleach features (Figure S7). These features are attributed to state-filling of conduction band electron levels [45] and can be correlated with exciton bands in the steady-state spectrum. The bleach centered at 530 nm, termed herein B3, corresponds to the bleach of the CdSe exciton transition [30,46]. The negative differential absorption with a minimum at 450 nm corresponds to two different bleach features. The sharp bleach feature at 450 nm (B1) corresponds to an exciton in the CdS rod. The shoulder at 470 nm (B2) reflects excitons located within the CdS rod, but in close proximity to the CdSe seed [30,46]. These bleach features were present in all samples when excited at 390 nm. With an increasing number of monolayers, all bleach features exhibit a bathochromic shift, e.g., the B3 bleach shifts from 530 nm (1 ML) over 545 nm (2 ML) to 567 nm (3 ML) (Figure 4), in agreement with the shifts observed for the CdSe-located lowest excitonic transition in the respective absorption spectra (Figure 1).

Next, changes in transient absorption spectra upon CdSe core excitation are discussed. For all samples, the spectral shape and position of the B3 bleach feature remains unchanged from CdS excitation, but with a higher relative amplitude. On the other hand, the nanorod composition affects the B1 and B2 features. To deliver a quantitative basis for the discussion, we subjected the transient spectra to a multi-Gaussian fitting model (Figure 4 and Table S3). For both 2 ML and 3 ML, the spectral position of the B1 feature remains the same even when exciting at the CdSe core. Again, this observation matches the expected quasi-type-II behavior: direct excitation of the CdSe core leads to the delocalization of the photo-generated electron over the entire CdS rod, which induces the B1, B2, and B3 features. For 1 ML, however, the B1 feature dominates less than B2 upon direct CdSe core excitation compared to CdS rod excitation. While we fitted the transient spectrum of 1 ML upon CdSe excitation with only one Gaussian in the spectral region of B1 and B2, the low signal-to-noise ratio may hide small contributions of the B1 feature, which are too minute to accurately fit. This change in the ratio of the B1 and B2 feature results in a red shift of the CdS bleach minimum from 450 nm (CdS excitation) to 460 nm (CdSe excitation). The change in spectral shape indicates the onset of a change in electronic structures in the 1 ML samples compared to the 2 ML and 3 ML samples. For the 1 ML sample, the electron appears to be more strongly confined to the CdS-CdSe interface region, i.e., in and around the CdSe seed, leading to decreased bleach in the B1 and stronger bleach of the B2 feature. This could be caused by increased density of trap states at the interface region with only one monolayer of CdS, and be indicative of an emerging type-I-like band structure [47,48].

## 3. Conclusions

It was originally expected that the 1 ML NRs, having the thinnest CdS shell, would display the highest H_2_ efficiency, as thinner shells may facilitate faster hole extraction. This prediction is in contrast to our findings here. We rationalize the lower H_2_ formation and hole extraction efficiencies of the 1 ML nanorods on the basis of stronger charge localization in the CdSe seed region as evidenced by time-resolved PL and excitation-wavelength-dependent transient absorption spectroscopy. Like in a classic case of type I band alignment, this localization corresponds with inferior charge separation within the semiconductor hybrid system due to stronger coulombic forces, which hinder transfer of these charges. Hence, the influence of shell width on the CdS band positions and hybrid bands alignment, in such quantum-confined structures, must be taken into consideration.

The 2 ML and 3 ML samples, on the other hand, exhibit true quasi-type-II band structure, with decreased overlap in electron and hole wavefunctions, and hence a decreased rate for radiative recombination that results in significantly longer PL lifetimes. The delocalization of the electron over the CdS shell supports efficient charge transfer to the electron shuttle, which is a prerequisite for H_2_ evolution. However, the improved efficiency for the critical hole transfer step, and the resulting superior catalytic activity of the 2 ML samples, can be explained by the lower shell thickness compared to the 3 ML sample, in accordance with the original design intention. Decreasing the shell width promotes quicker charge migration to the surface, leading to faster charge extraction and superior catalytic activity.

In summary, we successfully synthesized CdSe@CdS NRs with varying CdS shell width and studied their photocatalytic hydrogen evolution activity and relevant photophysical properties. The 2 ML NRs showed superior hole-quenching capabilities, in comparison to both 1 ML and 3 ML samples. This manifested into the highest H_2_ IQE of 92% (2 ML) under 405 nm excitation compared to 50% (3 ML) and 37% (1 ML). We attribute the improved efficiency to the enhanced hole transfer from the 2 ML sample, which outperforms the 3 ML sample thanks to its lower shell thickness and, accordingly, faster charge migration to the surface. For 1 ML, PL lifetime and transient absorption measurements revealed increased localization of charge carriers at the CdSe seed region compared to 2 ML and 3 ML. Similar to nanorods with type I band structure [2], this stronger charge confinement to the seed region and fast recombination results in lower photocatalytic performance compared to a true quasi-type-II band structure. The 2 ML structure presents an optimum shell thickness, which delicately balances between the two contradicting trends of faster hole transfer rates and stronger charge confinement with decreasing shell thickness. This work highlights the importance of fine-tuning nanomaterials at the atomic scale in order to precisely control their photophysical properties and maximize catalytic activity.

## Data Availability

The transient absorption data presented in this study is openly available in Zenodo at DOI:10.5281/zenodo.7074260.

## Supplementary Materials

The following supporting information can be downloaded at: https://www.mdpi.com/article/10.3390/nano12193343/s1; synthesis protocols, instrumentation utilized, setup and calculations used for the photocatalytic activity evaluation; Figure S1: Length and width distributions of 1, 2, and 3 ML NRs for an exemplary set; Figure S2: Absorption spectra of the rods reaction solution; Figure S3: Hydrogen evolution efficiencies of NRs during the first 24 h of the reaction; Figure S4: Hydrogen evolution efficiencies of NRs using 455 nm excitation light; Figure S5: Absorbance and photoluminescence spectrums of NRs in the presence of phenothiazine (PTZ) hole scavenger; Figure S6: PL lifetime of ODPA-capped NRs in toluene and MPA-capped NRs in water; Figure S7: Transient absorption spectrum of 1ML recorded at a delay time of 20 ps upon 390 nm excitation; Table S1: Fitting parameters used for the PL quenching of NRs with PTZ; Table S2: Multiexponential fitting parameters for PL lifetimes; Table S3: Multi-Gaussian fit of transient spectra at a delay time of 20 ps following either CdS rod or CdSe seed excitation. Reference [49] is cited in the supplementary materials.
