# Supplementary material for: The Other Dimension—Tuning Hole Extraction via Nanorod Width
Source: Nanomaterials (Basel). 2022 Sep 25;12(19):3343. doi: 10.3390/nano12193343 (PMC9565346; doi:10.3390/nano12193343)
Supplement: Supplementary file 1 [file nanomaterials-12-03343-s001.zip › nanomaterials-1929616-supplementary.pdf]

Supporting Information

The Other Dimension—Tuning Hole Extraction via Nanorod Width

Tal Rosner [1,†], Nicholas G. Pavlopoulos [1,†], Hagit Shoyhet [1,†], Mathias Micheel [2], Maria Wächtler [2,3,*], Noam Adir [1,*] and Lilac Amirav [1,*]

[1] Schulich Faculty of Chemistry, The Russell Berrie Nanotechnology Institute, The Nancy and Stephen Grand Technion Energy Program, Technion−Israel Institute of Technology, Haifa 32000, Israel
[2] Department Functional Interfaces, Leibniz Institute of Photonic Technology Jena, Albert-Einstein-Straße 9, 07745 Jena, Germany
[3] Institute of Physical Chemistry and Abbe Center of Photonics, Friedrich Schiller University Jena, Helmholtzweg 4, 07743 Jena, Germany
[*] Correspondence: maria.waechtler@leibniz-ipht.de (M.W.); noam@ch.technion.ac.il (N.A.); lilac@technion.ac.il (L.A.)
[†] These authors contributed equally to this work.

1. SYNTHESIS

Synthesis of CdSe Seeds

<u>CdSe Seeds used for the Synthesis of 1 and 2 ML NRs</u>

The CdSe synthesis was adopted with modifications from Carbone et al.[1] To a 25 mL three-neck round-bottom flask was added 0.060 g cadmium oxide (CdO), 3.00 g trioctylphosphine oxide (TOPO), 0.208 g octadecylphosphonic acid (ODPA), and a ½″ Teflon-coated stir bar. The reaction solution was heated to 150°C under a vacuum for 2 hours. The reaction was then put under nitrogen, and the solution was heated to 350 °C at 300 RPM to dissolve the CdO until it turned optically clear and colorless. During this process, the flask was shaken as necessary to dissolve all CdO that may have been adhered to the walls of the flask. At this point, 1.80 mL of trioctylphosphine (TOP) was injected into the flask, and the temperature was raised to 396 °C. Upon reaching 396 °C, the flask was removed from the heating mantle. Once the solution had cooled down to 350 °C, 0.43 mL of the TOP=Se stock solution was rapidly injected from a 1 mL syringe equipped with a 22 gauge 1 ½ inch needle. The reaction mixture was allowed to cool to room temperature, and subsequently, 5.0 mL of anhydrous toluene was injected to prevent the solidification of the high-boiling-point solvent. The solution was purified with three centrifugation rounds of ethanol and toluene. The final pellet was dissolved in toluene.

<u>CdSe Seeds used for the synthesis of 3 ML NRs</u>

This procedure modifies the above protocol to attain CdSe QDs of the same size (2.3 nm) but with different crystal structures. This is attained by varying the concentration of ligands (ODPA) and the injection temperature of the selenium precursor (TOP=Se). These modifications affect the growth kinetics of the Cd^{2+} and Se^{2-} monomers into CdSe QDs, and ligand adsorption and desorption rate constants along the nanoparticle surface during the growth phase.

The following synthetic modifications were made to Section 2.1:

- Using 0.280 g ODPA instead of 0.208 g ODPA
- Injection of 1.50 ml TOP instead of 1.80 ml TOP
- Injection of 0.35 ml TOP=Se at a stable 370 °C instead of injection of 0.43 ml TOP=Se at 350° C while the mantle is cooling

Synthesis of CdSe@CdS Nanorods

A well-known CdSe@CdS NR synthesis was adopted from Carbone et al.[26] To a flame-dried 25 mL three-neck round-bottom flask was added CdO (0.075 g). Subsequently, TOPO (3.0 g), ODPA (0.290 g), and HPA (0.080 g) were loaded to the 25 mL three-neck-round bottom. The flask was then equipped with a reflux condenser and a 1/2" Teflon-coated stir bar. The two necks of the flask were sealed with Teflon-tape coated rubber septa, and a thermocouple was inserted through the right-hand neck of the flask after a single puncture with a 21 Ga needle. After pumping the flask to a vacuum for 90 minutes at 150 °C (three evacuation and backfilling steps, average pressure ~ 0.77 torr), the resulting solution was heated to above 300°C under argon until clear and colorless. This took approximately 10 minutes above 300 °C for complete complexation to occur, including shaking. The flask was then cooled to 150 °C and exposed to three evacuation/backfill cycles (10 minutes each). Next, the flask was heated to 350 °C. Above 300 °C, TOP (1.8 mL) was injected, after which the temperature was allowed to recover to 350°C and stabilize for 30 before injection of the solution of sulfur precursor + nanocrystals (1.8 mL of stock was quickly injected into the flask one 6 mL syringe). The nanocrystals were allowed to grow for a total of 6 minutes, after which the heating mantle was removed. Toluene (~10 mL) was injected after cooling to < 110 °C to prevent solidification of the high boiling point solvent. The solution was purified with three centrifugation rounds of ethanol and toluene. The final pellet was dissolved in toluene.

MPA Ligand Exchange

NRs were precipitated from toluene, redispersed in 3.0 ml chloroform, and then placed into a 40 ml scintillation vial equipped with ½" stir. Next, 3.0 ml of HPLC H_2O was added to create a biphasic layer. Next, 0.50 ml of MPA and 0.50 ml of a KOH/H_2O solution (1g/ml concentration) were added dropwise. The solution was stirred overnight to initiate the ligand exchange. At the end of the exchange, the nanorod solution (now in H_2O) was mixed with ethanol and centrifuged for 30 minutes at 10000 RPM. This step was repeated once. At the end of the centrifugation steps, the supernatant was removed, and the precipitate was dissolved in 3.0 ml of water.

Synthesis of Platinum Nanoparticles

To a 100 mL round-bottom flask was added an H_2PtCl_6 stock solution (10 mL, 1.2 g/100 mL – 0.12 g H_2PtCl_6) and stirred at 300 RPM in an oil bath at 110 °C until the solution began to reflux. Upon refluxing, the flask was quickly removed from the heat, followed by sodium citrate tribasic dehydrate addition (as a reducing agent). Subsequently, a poly(acrylic acid) stock solution was added to the solution to serve as a proton conducting ligand. The solution was then heated to reflux for 1 hour, over the course of which a dark brown solution was obtained (color began to charge ~ 30 minutes in). Post reaction, the sample was passed through a 1-micron syringe filter to obtain a deep brown tinted solution that was completely homogeneous. To remove excess citrate anions, the sample was stirred over anion exchange resin (Amberlite MB-1 ~ 0.25 g/mL) for 20 minutes, followed by being passed through a syringe filter (1 micron) again.

2. INSTRUMENTATION

Transmission Electron Microscopy (TEM). Transmission electron microscopy (TEM) was conducted to quantify the length, width, morphology, and monodispersity of the NRs. TEM was conducted via the FEI Tecnai G2 T20 S-Twin TEM, running at 200keV with a LaB_6 electron source. Nanorod solutions were drop-casted onto a carbon-coated copper grid (Cu, square, 200 mesh) and vacuumed for at least 24 hours before being inserted into the microscope.

X-ray Diffraction (XRD). XRD was carried out with a Rigaku SmartLab X-ray diffractometer using Cu Kα radiation (wavelength, 0.154 nm) with a slit of 2 mm.

UV/Vis Spectroscopy. UV–Vis absorption spectroscopy was performed using an Agilent Cary 5000 UV–Vis-NIR spectrophotometer using standard 10mm fluorimeter cuvettes. Spectra were studied using the accompanying Cary WinUV software package.

Photoluminescence (PL). PL measurements were conducted using a Horiba Jobin Yvon Fluorolog 3 spectrometer. The samples were excited with a 405 nm LED. Spectra were studied using accompanying Origin software. For PL quenching measurements, PL spectra were recorded on an Edinburgh FS5 spectrofluorometer. The excitation wavelength was set to 415 nm to avoid absorption of the excitation light by phenothiazine (PTZ), as PTZ has tail absorption down to ~405 nm. All PL scans were taken at a 1 nm step size with a 0.3 sec dwell time and slit widths of 1 nm for both excitation and emission. PTZ used for the quenching experiments was stored in the glovebox, and the stock solution for quenching was made entirely in the glovebox. For each nanorod sample, quenching was performed with a freshly prepared stock solution, and the nanorod solution was fully deaerated with argon. The approximate time between the preparation of

the sample to performing the quenching measurements was the same in each case (~5 minutes). All samples were kept air-free during quenching measurements to minimize interactions with O_2.

Photoluminescent Lifetime. Time-resolved photoluminescence measurements were conducted using an Edinburgh Instruments Life Spec II time-correlated single photon counting (TCSPC) system utilizing a 405 nm laser for excitation and a 1 μs pulse duration.

Transient Absorption (TA). The fs time-resolved measurements were performed on a system consisting of a Ti: sapphire amplifier (Legend-Elite, Coherent Inc., Santa Clara, CA, USA), producing pulses centered at 795 nm with a repetition rate of 1 kHz and a pulse duration of <100 fs. The pump pulses centered at 390 nm were generated by second harmonic generation by focusing the fundamental into a barium borate (BBO) crystal, and other excitation wavelengths were generated in a collinear optical parametric amplifier (TOPAS-C, LightConversion Ltd.) A white light continuum with a spectral range from 350 to 750 nm was generated by focusing a fraction of the fundamental into an eccentrically rotating CaF_2 crystal to probe the sample. The pump pulses were delayed with respect to the probe pulses by means of an optical delay stage (maximum delay: 2 ns) and focused into the sample by a lens (f = 75 cm). The repetition rate of the pump pulses was reduced to 500 Hz by a mechanical chopper, and the polarization of the pump with respect to the probe pulses was set to the magic angle (54.7°) using a Berek compensator (Thorlabs GmbH, Bergkirchen, Germany) and a polarizer. The optical density of the sample was set to 0.4–0.6 at 390 nm for all measurement conditions. The power density of the pump pulse at the sample position was adjusted to 0.006 $W \cdot cm^{-2}$ and below for 390 nm excitation and, because of the much lower CdSe absorption and to ensure a similar number of absorbed photons, to 0.1 $W \cdot cm^{-2}$ for measurements exciting the CdSe core. The white light continuum was split into probe and reference. The probe pulse was focused onto the sample by a concave mirror (f = 500 mm) and spatially overlapped with the pump pulse. The probe and reference were collected by a detection system (Pascher Instruments AB, Lund, Sweden) consisting of a spectrograph (Acton, Princeton Instruments, Trenton, NJ, USA) equipped with a double-stripe diode array detector. The diode array is read out with the laser repetition rate, and the signal (ΔA) is calculated from two consecutive probe pulses, corresponding to pump-on and pump-off conditions. Due to the high laser power when exciting the CdSe core, the probe light after the sample was filtered by using a polarization filter to reduce scattered pump light as much as possible. The samples were prepared and measured under inert conditions using home-built sealed, airtight cuvettes with 1 mm path length. Recorded transient spectra were chirp corrected using the KiMoPack software.[49]

3. PHOTOCATALYTIC HYDROGEN EVOLUTION

Experimental Setup

In the reaction chamber, a solution containing MPA-exchanged nanorods, 0.8 mM Pt-NPs, 0.11 M MPA, and 4.5 mM MV^{2+} was dispersed in a phosphate buffer (pH 6.2, 50 mM) and placed inside a custom-built gas-tight reaction cell. The cell was closed with a butterfly clamp and purged with argon (99.999%) flowing at a rate of 10 ml/min through the cell and into a gas chromatography (GC-TCD, Agilent 7890A Series with thermal conductivity detector (TCD)). The cell was purged with argon until oxygen and nitrogen could not be detected. A Thorlabs LED (5 mW, 405 or 530 nm) was switched on to illuminate the solution.

Internal Quantum Efficiency Calculations

<u>Photon Flux Calculation</u>

The photon flux was calculated by measuring the LED power (which was adjusted to the desired value and measured using a Thorlabs Digital Optical Meter – PM206), assuming all photons had the same wavelength of 405nm (4.908×10^{-19} Joule/photon). In this adjustment of the power, we accounted for the irradiated area over the sample, as well as for the absorption of the reaction cell window and other minor losses in the setup. The LED wavelength (λ) we used was 405 nm; therefore, the energy each photon carries:

$$E(Photon) = \frac{h \times c}{\lambda} = \frac{6.626 \times 10^{-34}[\,j \times sec] \times 3 \times 10^{8} \left[\frac{m}{sec}\right]}{405 \times 10^{-9}\,[m]} = 4.908 \times 10^{-19} \left[\frac{j}{Photon}\right]$$

The LED power was set to 5 mW. Since the absorbance of the nanorods and platinum nanoparticles is each 1.0, the nanorods absorb half the power (or 2.5 mW). Thus, the photon flux (number of photons per second) is given by:

$$Power = \frac{2.5 \times 10^{-3} \left[\frac{j}{sec}\right]}{4.908 \times 10^{-19} \left[\frac{j}{Photon}\right]} \quad = \quad 5.09 \times 10^{15} \left[\frac{Photons}{sec}\right]$$

Internal Quantum Efficiency Calculation

The GC measurement results with an area for the H_2 peak, which is converted using the calibration to the H_2 production flow rate. Using the production flow rate, Q [mL/sec], and the ideal gas law, we can calculate the number of hydrogen moles:

For ideal gas: 1 mol gas (@21°C, the temperature of the lab) = 24.1 L

Thus, the flow rate with units of mL/sec can be converted to a number of moles and later on using Avogadro number to molecules/sec. At 5 mW of power, we obtained a flow of 5.63 μL/min, which is equivalent to:

$$\frac{5.63 \times 10^{-6} \left[\frac{L}{min}\right] \times \frac{1}{60} \left[\frac{min}{sec}\right]}{24.1 \left[\frac{L}{mol}\right]} \quad = \quad 3.89 \times 10^{-9} \left[\frac{mol}{sec}\right]$$

Next, we can calculate the production rate as the number of hydrogen molecules per sec:

$$3.89 \times 10^{-9} \left[\frac{mol}{sec}\right] \times 6.022 \times 10^{23} \left[\frac{H_2}{mol}\right] = 2.34 \times 10^{15} \left[\frac{H_2}{sec}\right]$$

Alternatively, the calibration with the electrolysis cell can yield the number of hydrogen molecules per sec directly. Each hydrogen molecule production requires two electrons and, therefore, two photons. Thus, the internal quantum efficiency of the sample is defined as IQE = $2N_{H2}/N_{hv}$

$$IQE = \frac{2 \times 2.34 \times 10^{15} \left[\frac{H_2}{sec}\right]}{5.09 \times 10^{15} \left[\frac{Photons}{sec}\right]} \quad = \quad 0.92$$

4. ADDITIONAL DATA AND FIGURES

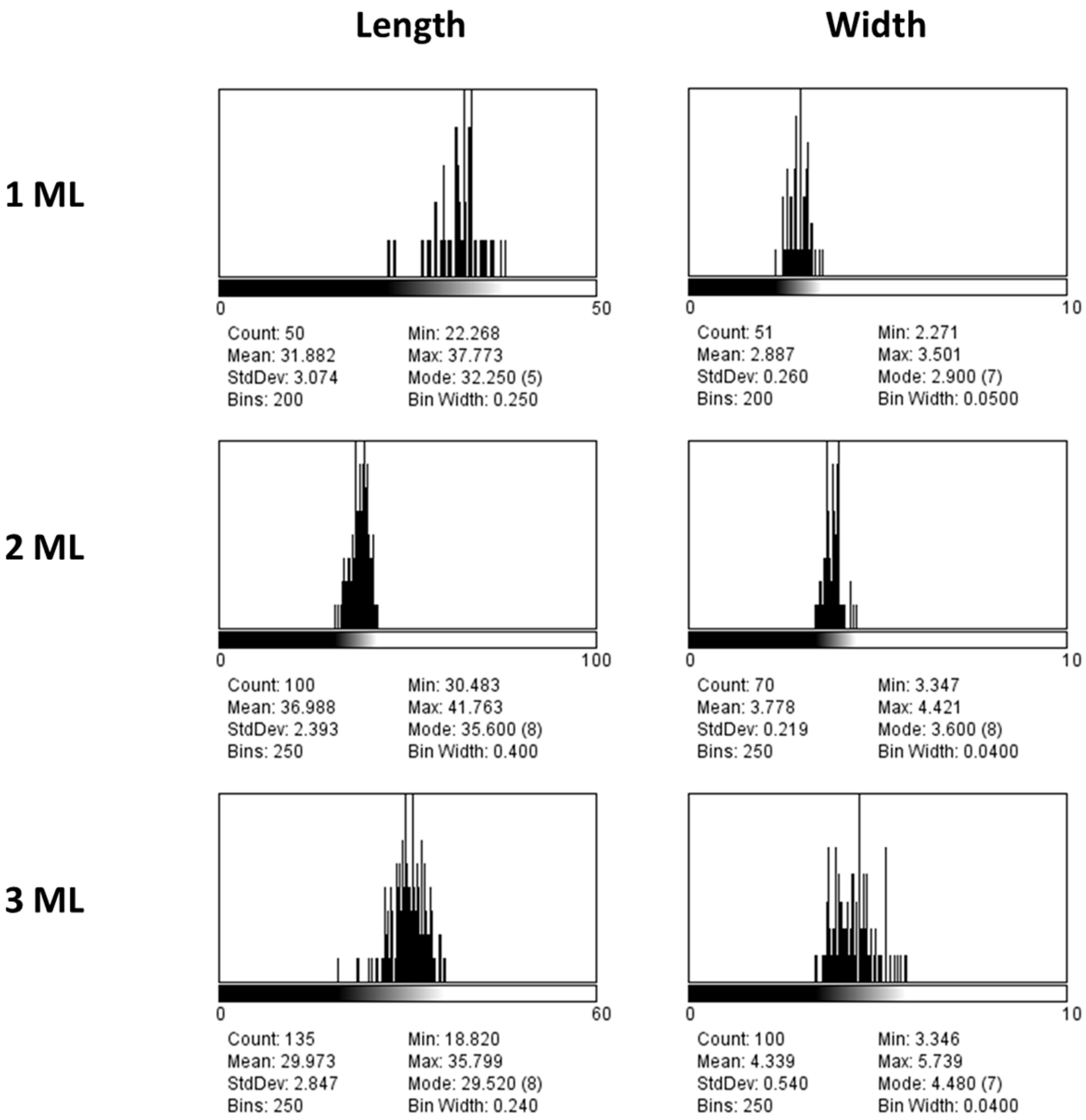

Figure S1. Length and width distributions of 1,2, and 3 ML NRs for an exemplary set.

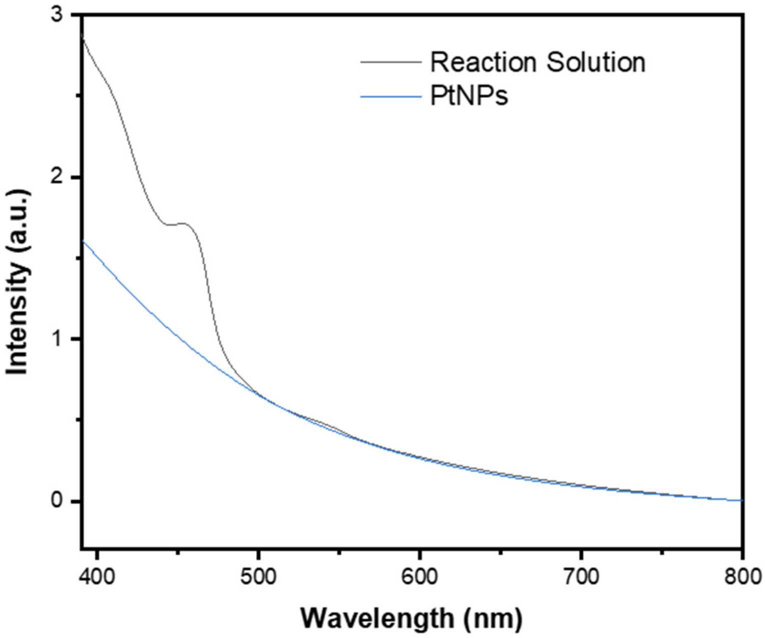

Figure S2. Absorption spectra of the rods reaction solution (2 ML NRs, PtNPs, MV^{2+}, MPA, pH 6.2 phosphate buffer) and PtNPs. .

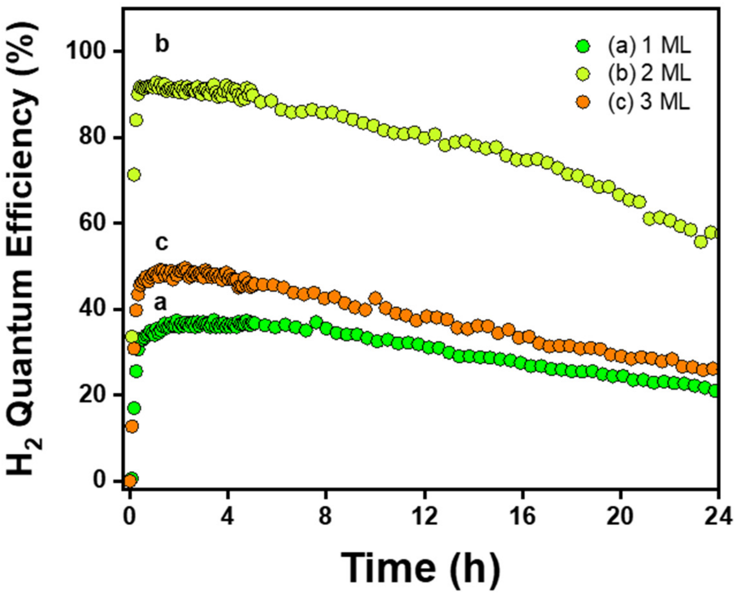

Figure S3. Hydrogen evolution efficiencies of NRs during the first 24 hours of the reaction.

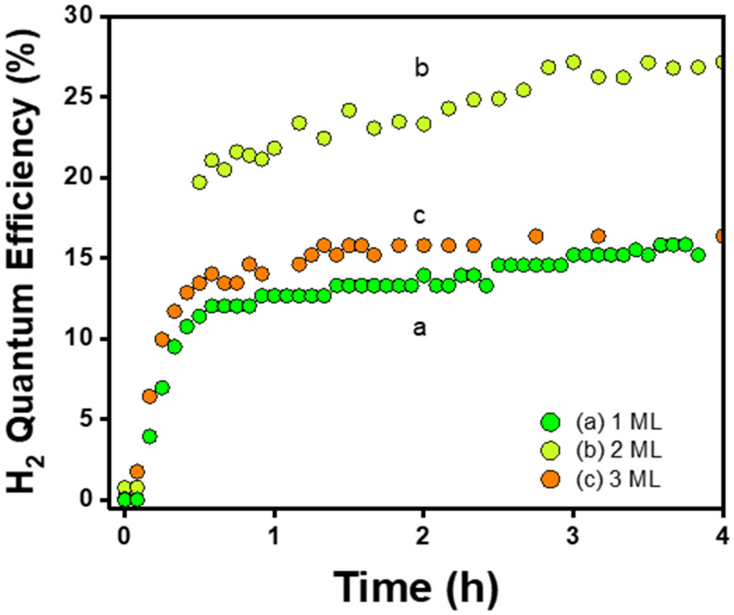

Figure S4. Hydrogen evolution efficiencies of NRs using 455 nm excitation light.

Photoluminescence quenching with Phenothiazine

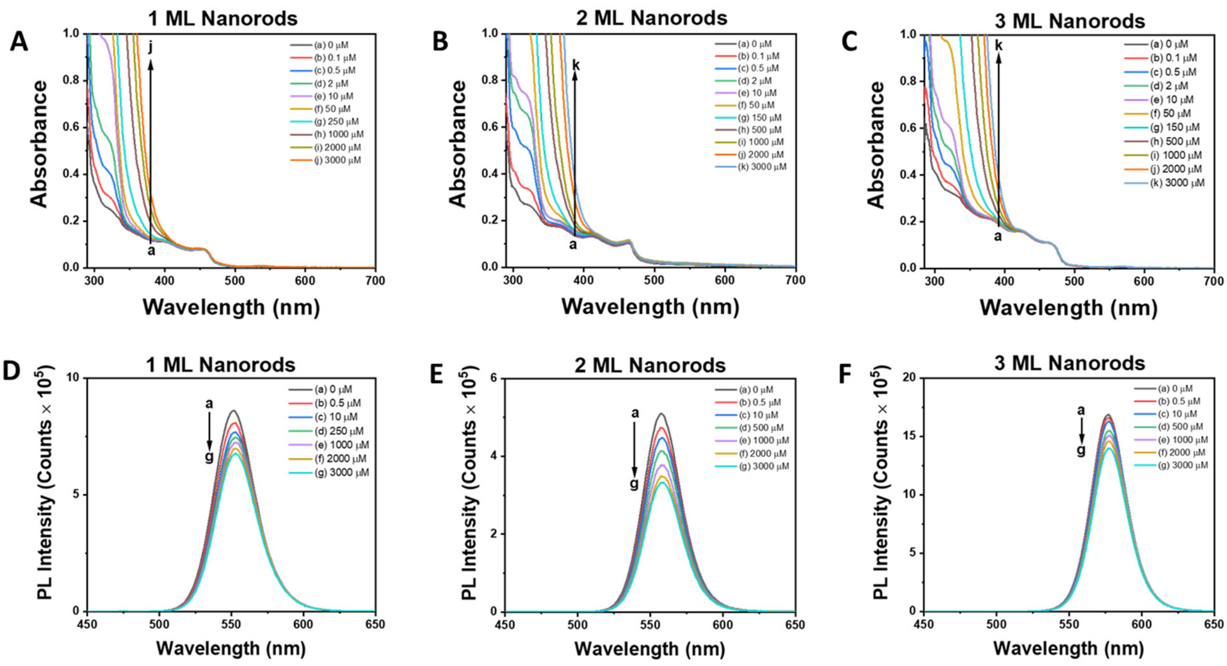

Figure S5. (**A**–**C**) Absorbance and (**D**–**F**) photoluminescence spectrums of NRs in the presence of phenothiazine (PTZ) hole scavenger. Rising absorbance below 400 nm is caused by the increasing concentrations of PTZ (Figure S5 **A**–**C**). Decreasing photoluminescence intensity indicated transfer of holes to PTZ (Figure S5 **D**–**F**).

Table S1. Fitting parameters used for the PL quenching of NRs with PTZ. PL quenching data were fitted using the following multi-site binding equation, where B_1 and B_2 represent the relative number of each type of binding site, k_1 and k_2 are the binding constant for the quencher at those sites, and [Q] is the quencher concentration.

Sample	F_1 (%)	K_1 (M^{-1})	F_2 (%)	K_2 (M^{-1})
**1 ML NRs**	12 ± 1.4	$1.37 \times 10^6 \pm 0.67 \times 10^6$	88 ± 0.7	41.5 ± 5.36
**2 ML NRs**	15 ± 2.4	$1.25 \times 10^6 \pm 0.86 \times 10^6$	85 ± 1.1	111.2 ± 9.96
**3 ML NRs**	5 ± 0.9	$3.47 \times 10^6 \pm 0.14 \times 10^6$	95 ± 0.6	51.6 ± 4.11

$$\frac{I}{I_0} = \frac{\mathrm{F}_1}{1 + \mathrm{K}_1[\mathrm{Q}]} + \frac{\mathrm{F}_2}{1 + \mathrm{K}_2[\mathrm{Q}]}$$

PL Lifetime of NRs Capped with ODPA and MPA Ligands

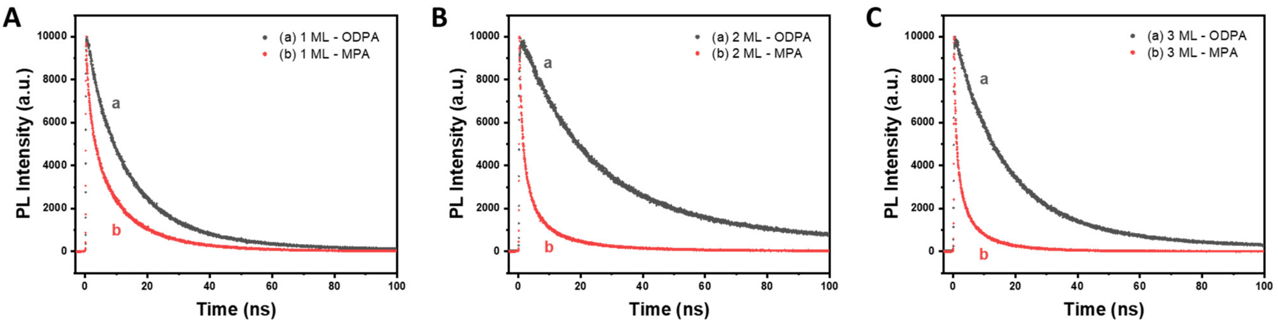

Figure S6. PL lifetime of ODPA-capped NRs in toluene and MPA-capped NRs in water. Samples were placed in a 10 mm cuvette and were excited with a 405 nm laser for PL decay measurements.

Table S2. Multiexponential Fitting parameters for PL lifetime of NRs with ODPA and MPA ligands.

Sample	A_1	τ_1 (ns)	A_2	τ_2 (ns)	A_3	τ_3 (ns)	τ_{avg} (ns)
1 ML NRs - ODPA	6011 ± 49	8.9 ± 0.1	4305 ± 51	22.3 ± 0.1	-	-	17.5 ± 2.3
1 ML NRs - MPA	3270 ± 30	1.5 ± 0.1	4116 ± 35	6.9 ± 0.1	2475.8 ± 56	17.8 ± 0.2	12.8 ± 1.5
2 ML NRs - ODPA	7596 ± 53	19.8 ± 0.1	2598 ± 51	63.8 ± 1.1	-	-	42.9 ± 2.3
2 ML NRs - MPA	6584 ± 21	1.0 ± 0.1	3823 ± 17.8	4.8 ± 0.1	1021 ± 12	29.2 ± 0.1	11.2 ± 1.6
3 ML NRs - ODPA	8235 ± 39	14.5 ± 0.1	2049 ± 40	41.6 ± 0.5	-	-	25.8 ± 2.1
3 ML NRs - MPA	5747 ± 39	**0.8** ± 0.1	3481 ± 21	**3.7** ± 0.1	1208 ± 17	12.7 ± 0.1	7.5 ± 1.6

PL lifetime data were fit using the following multiexponential decay equation:

$$\mathrm{I} = \sum_i A_i \exp(-x/\tau_i)_{\ i}$$

Average PL lifetime was calculated using the following equation:

$$\tau_{avg} = \frac{\sum A_i\,\tau_i^2}{\sum A_i\,\tau_i}$$

Transient absorption

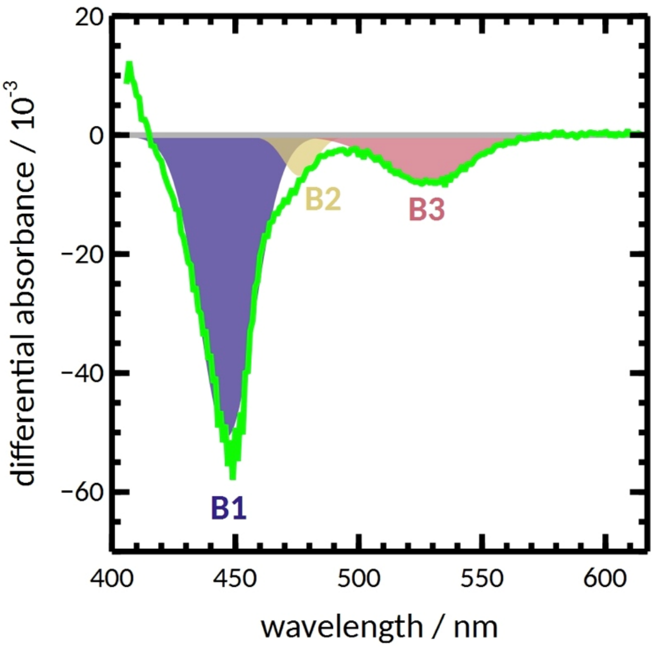

Figure S7. Transient absorption spectrum of 1ML recorded at a delay time of 20 ps upon 390 nm excitation. The three bleach features B1(corresponding to CdS rod transitions), B2 (CdS rod near the CdSe interface), and B3 (CdSe seed) have been highlighted.

Transient spectra were subjected to a multi-Gaussian fit using Origin (v8.1) software using five Gaussians: one, respectively, for the bleach features, B3 and B2, two for the bleach feature, B1, and one positive one for the photoinduced absorption at higher energies than B1. This approach is in good agreement with our earlier observation that the lowest energy CdS nanorod steady-state absorption can be well described by two Gaussians, whereas the bulb and CdSe features only require one each.[40] The fit was performed after transforming wavelengths to photon energies.

Table S3. Multi-Gaussian fit of transient spectra at a delay time of 20 ps following either CdS rod or CdSe seed excitation. For the following table, the central wavelengths λ_c of each Gaussian are indicated. Data marked with an asterisk (*) have been determined byfixing this central wavelength for the fit.

Assignment	1ML		2ML		3ML	
	λ_c(CdS) / nm	λ_c(CdSe) / nm	λ_c(CdS) / nm	λ_c(CdSe) / nm	λ_c(CdS) / nm	λ_c(CdSe) / nm
**B3**	526 ± 1	533 ± 11	545 ± 6	546 ± 2	567 ± 1	569 ± 1
**B2**	469 ± 6	462 ± 1	479 ± 12	480 ± 1	503*	503 ± 1
**B1 - 1**	449 ± 1	–	467 ± 6	465 ± 2	462 ± 1	473 ± 1
**B1 - 2**	441 ± 3	–	457 ± 7	450 ± 3	447 ± 1	459 ± 1

References

1. Müller, C., Pascher, T., Eriksson, A., Chabera, P.; Uhlig, J. KiMoPack: A python Package for Kinetic Modeling of the Chemical Mechanism. *J. Phys. Chem. A* **2022**, *126*, 4087–4099.
